# Supplementary material for: HD-Zip III Gene Family: Identification and Expression Profiles during Leaf Vein Development in Soybean
Source: Plants (Basel). 2022 Jun 29;11(13):1728. doi: 10.3390/plants11131728 (PMC9269512; doi:10.3390/plants11131728)
Supplement: Supplementary file 1 [file plants-11-01728-s001.zip › plants-1765677-supplementary.pdf]

plants

MDPI

Article

HD-Zip III Gene Family: Identification and Expression Profiles During Leaf Vein Development in Soybean

Jing Gao [1,2,†], Jiyu Chen [1,2,†], Lingyang Feng [1,2], Qi Wang [1,2], Shenglan Li [1,2], Xianming Tan [1,2], Feng Yang [1,2,*] and Wenyu Yang [1,2]

Supplementary Materials

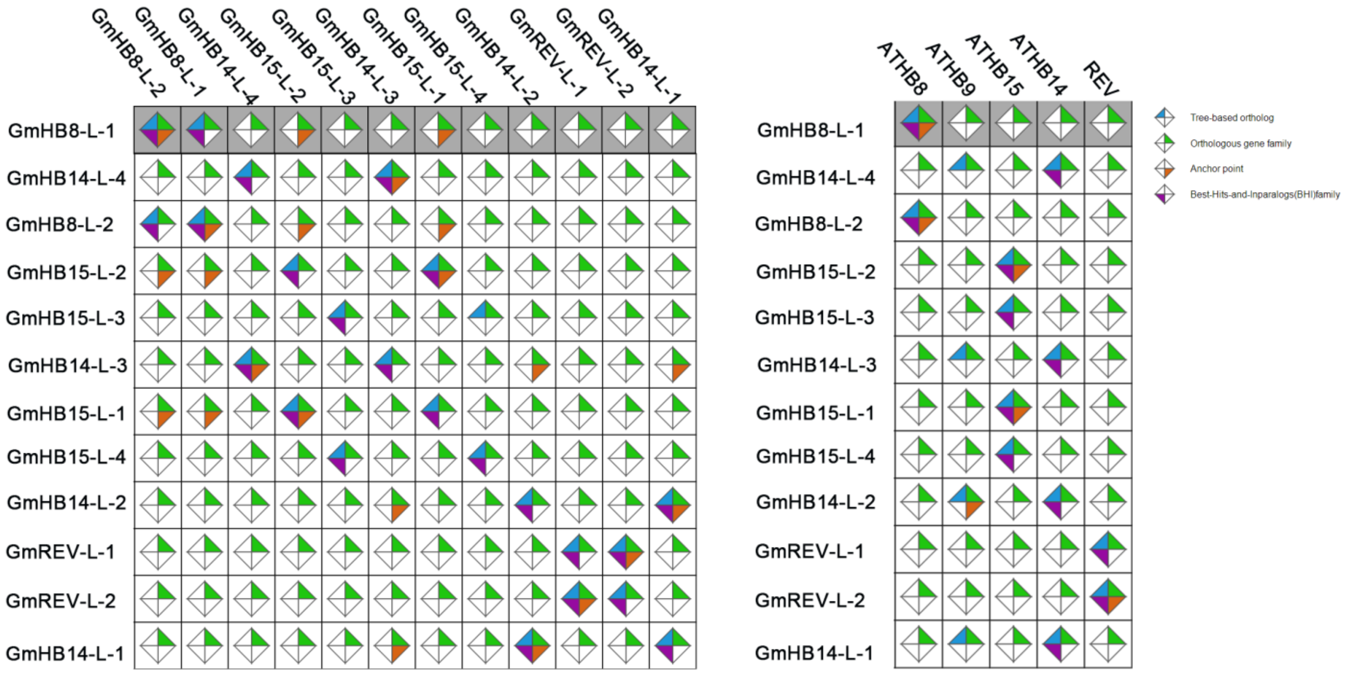

Figure S1. Orthologous genes between soybean and Arabidopsis. List of orthologous gene pairs obtained from PLAZA.

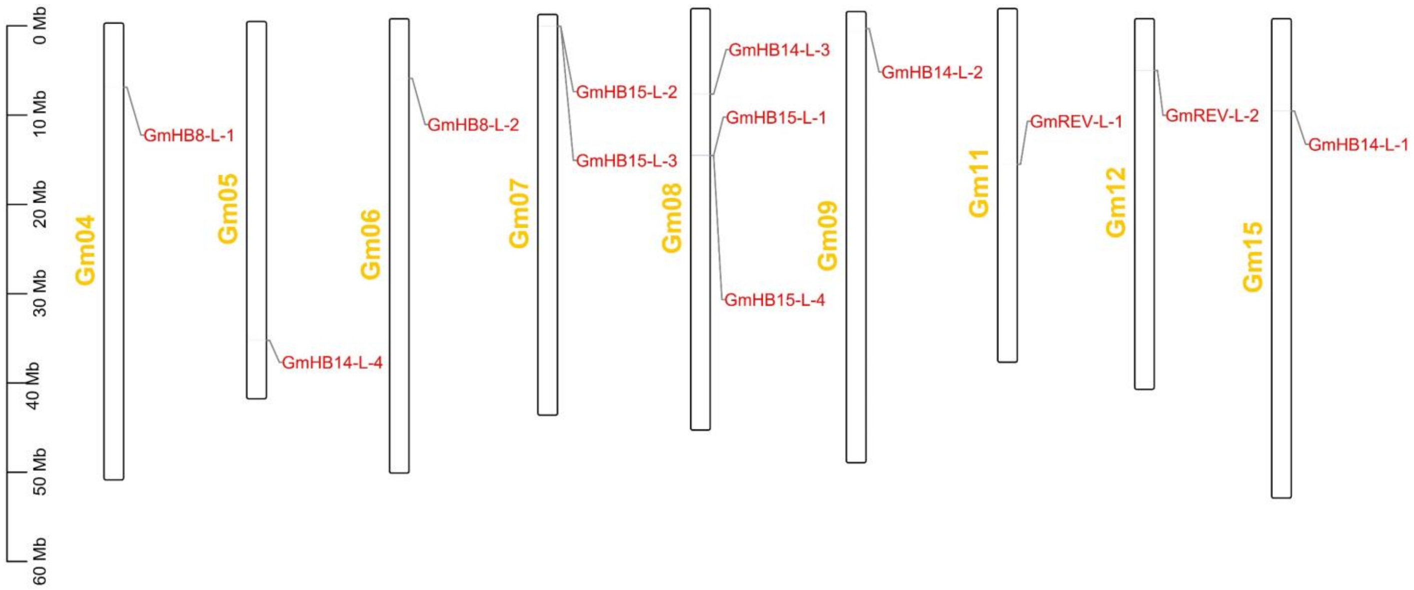

Figure S2. Chromosomal distributions of the identified soybean HD-Zip III genes. Chromosomal locations are shown from top to bottom on the corresponding chromosomes. The bar on the left side indicates chromosome sizes in megabases. The scale represents chromosome length.

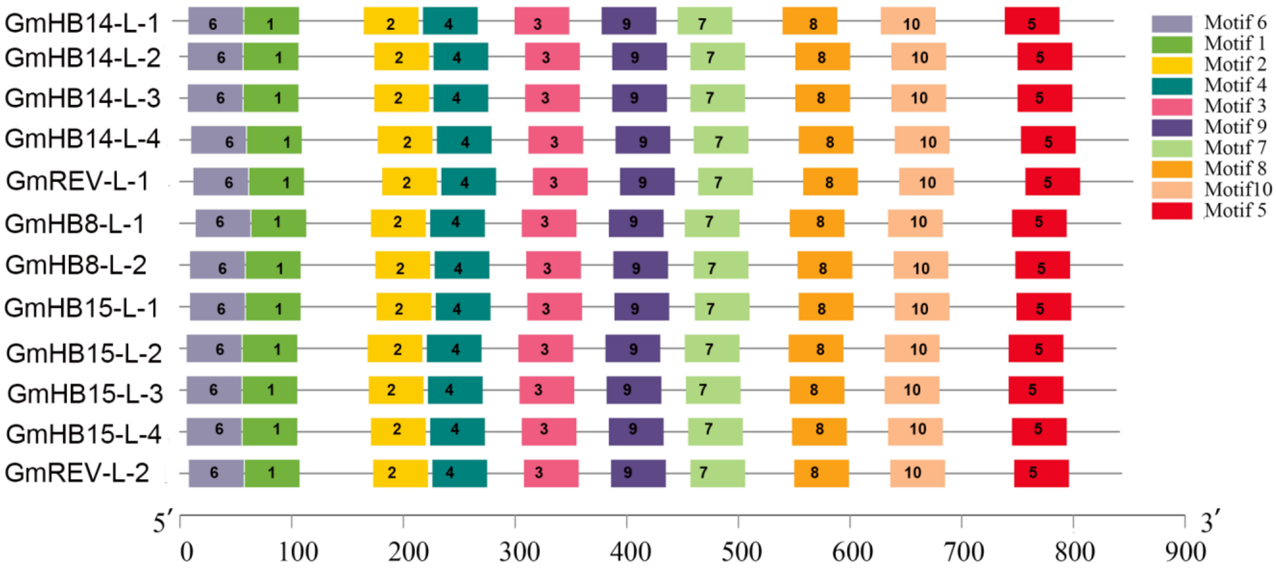

Figure S3. Motif of the 12 GmHD-Zip III proteins. Different colored boxes stand for different motifs. The length of each box in the figure represents the actual size of the motif in the proteins.

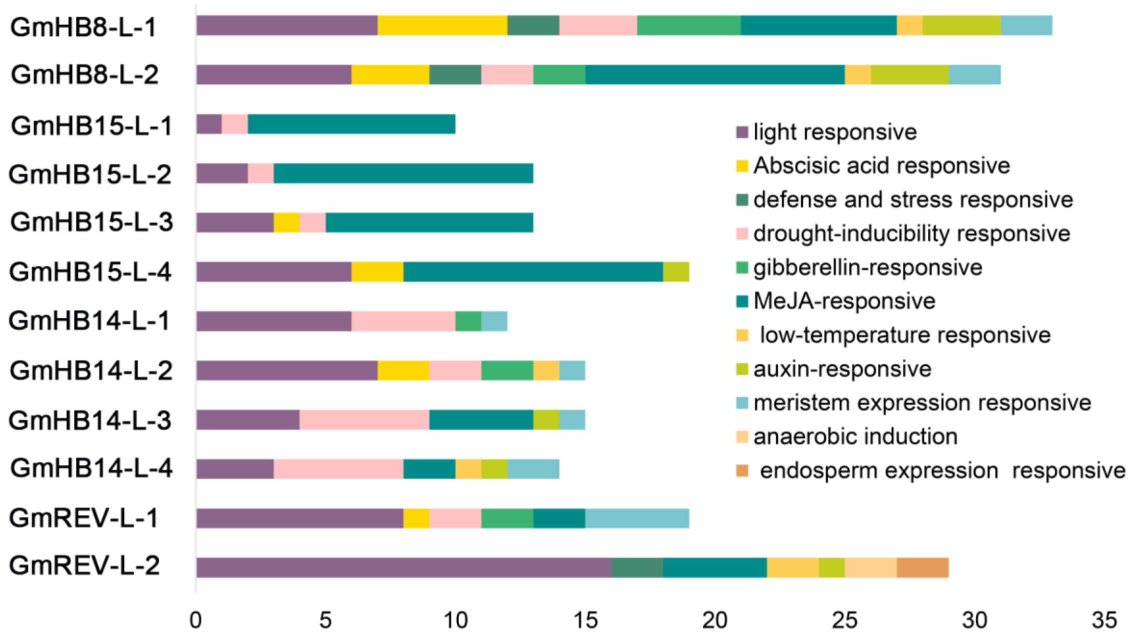

Figure S4. Analysis of cis-acting elements in the promoter regions of GmHD-Zip III family genes.

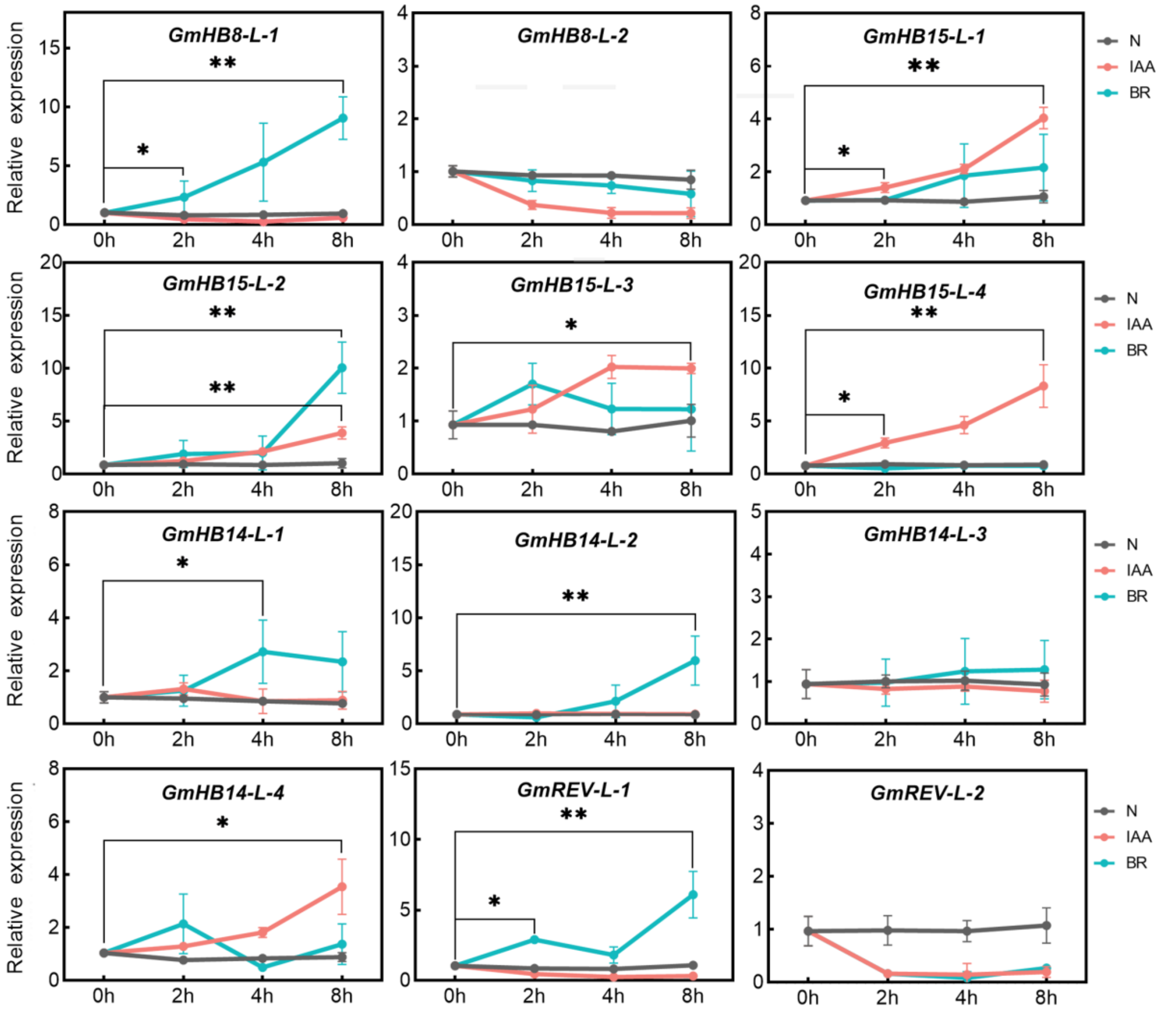

Figure S5. Expression profiles of GmHD-Zip III under IAA and BR treatment. Asterisks on top of the bars indicate statistically significant differences (*P < 0.05, **P < 0.01).

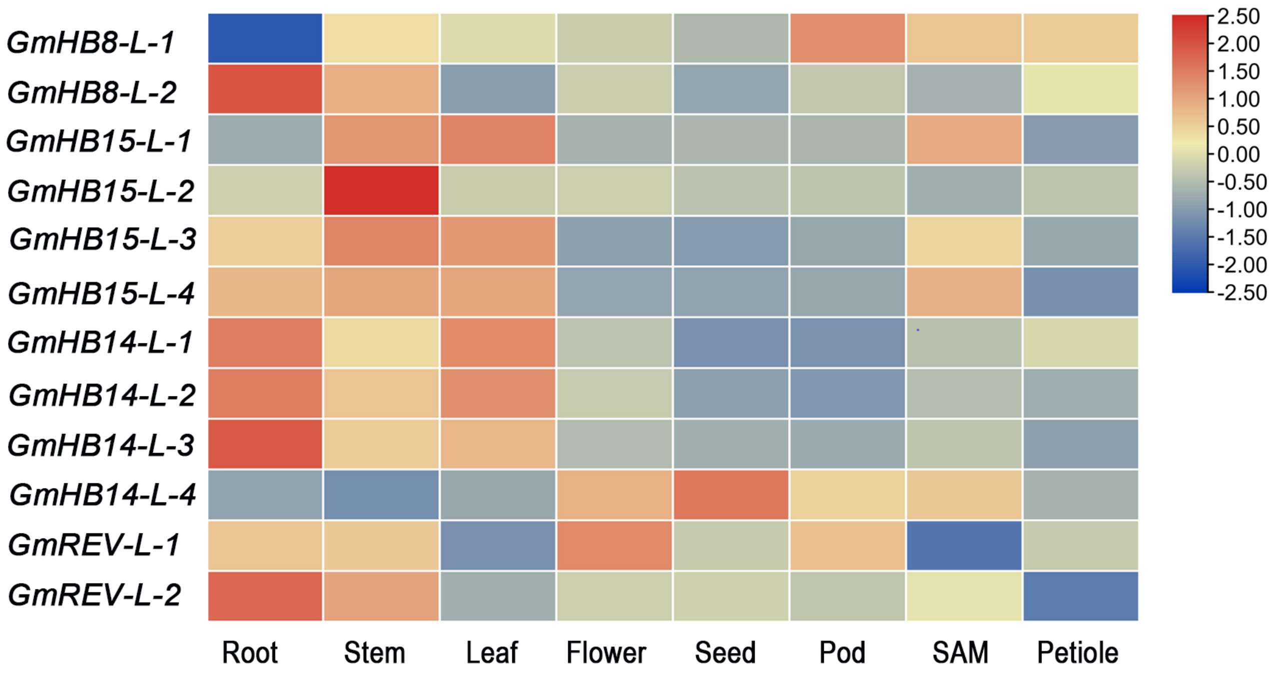

Figure S6. Expression profiles of *GmHD-Zip III* genes in eight soybean tissues in reads/Kb/million. The normalized values of gene expression were log2-transformed and visualized in the form of heatmaps.

Table S1. Primers sequences used in qRT-PCR.

GeneID	Forward primer (5′→3′)	Reverse primer (5′→3′)
GLYMA_04G085000	GAGTGGGTCCAAATGCCTGG	CTAGACCAACAAGGCCGCAG
GLYMA_06G086600	ACTCAGCTCATTCTGGGAGC	GTGAGAACCAAAGCGCGAAG
GLYMA_08G201900	GCTGCCTAGTGGGTACCTGA	CAGTGGGCGTAGCACTTCTG
GLYMA_07G016500	ACACCCAGATTACTACGCAGG	TTATCAAATTGTGCTGCTGACCAC
GLYMA_07G016700	GCCTGGAATGAAGCCTGGTC	CCTGAAACCACAAAGGCCGA
GLYMA_08G202000	TTCGCCGCCTGCAACTCATTC	ACGACTCCTTCCTCTGCTTCTCTC
GLYMA_15G129700	GTCTCTTCCTGGTTCGCCTG	AGCACATGATTGCATCGGGA
GLYMA_09G023600	TCTTTTCAGGGTTTTGCATCTCTC	ACATGAAGGCTAGGCAGTGAT
GLYMA_08G124400	AGTGGTCAACGCCAACATCA	AGTAGCTTTTGAAAGGAACGCC
GLYMA_05G166400	ACAGGTGGAGGCTTTGGAGAGG	TCACGACATCTGCGATTCTGGAAC
GLYMA_11G145800	TTGCTACAAATCGGACCCTGG-3'	CACACTGCGGACATATTGGC-3'
GLYMA_19G147900	TGCACAGAAAGTGCTTCTAAACTG	GCTTCCAGACAAATCGTGATGA
GLYMA_12G075800	AGTGCCCTAAGCCTAGTTCTCTGAG	AGTAGTTGCTGCTGATGGAGTATGC

Table S2. The homologous proteins of HD-ZIP III in legumes.

Name	Scientific Name	Accession	E value	Query Cover
AhHB8-L-1	*Arachis hypogaea*	XP_025681305.1	0	0.98
AhHB8-L-2	*Arachis hypogaea*	XP_025623723.1	0	0.98
AhHB15-L-1	*Arachis hypogaea*	XP_025674176.1	0	0.98
AhHB15-L-2	*Arachis hypogaea*	XP_025610204.1	0	0.98
AhHB15-L-3	*Arachis hypogaea*	RYR49685.1	0	0.98
AhHB8-L-3	*Arachis hypogaea*	RYQ85287.1	0	0.98
AhREV-L-1	*Arachis hypogaea*	XP_025685402.1	0	0.99
AhREV-L-2	*Arachis hypogaea*	QHN98691.1	0	0.99
AhHB14-L-1	*Arachis hypogaea*	XP_025646247.1	0	0.99
AhHB14-L-2	*Arachis hypogaea*	XP_025694278.1	0	0.99
AhREV-L-3	*Arachis hypogaea*	RYR23266.1	0	0.99
AhHB8-L-4	*Arachis hypogaea*	RYR33648.1	0	0.78
AhREV-L-4	*Arachis hypogaea*	QHO30808.1	0	0.99
AhREV-L-5	*Arachis hypogaea*	XP_025614062.1	0	0.99
AhREV-L-6	*Arachis hypogaea*	XP_025670948.1	0	0.99
AhREV-L-7	*Arachis hypogaea*	RYR42882.1	0	0.99
AhREV-L-8	*Arachis hypogaea*	RYQ97997.1	0	0.99
CaHB15-L-1	*Cicer arietinum*	XP_004510296.1	0	0.99
CaHB8-L-1	*Cicer arietinum*	XP_004501146.1	0	0.98
CaHB15-L-2	*Cicer arietinum*	XP_004486776.1	0	0.98
CaHB14-L-1	*Cicer arietinum*	XP_004486450.1	0	0.99
CaREV-L-1	*Cicer arietinum*	XP_004505999.1	0	0.99
CaHB14-L-2	*Cicer arietinum*	XP_004504217.1	0	0.99
GmHB8-L-1	*Glycine max*	XP_003522716.1	0	0.98
GmHB8-L-2	*Glycine max*	XP_003526496.1	0	0.98
GmHB15-L-1	*Glycine max*	XP_003531652.1	0	0.99
GmHB15-L-2	*Glycine max*	XP_003530109.1	0	0.99
GmHB15-L-3	*Glycine max*	XP_003530112.1	0	0.99
GmHB15-L-4	*Glycine max*	XP_003531653.1	0	0.98
GmHB14-L-1	*Glycine max*	XP_003546255.1	0	0.99

GmHB14-L-2	*Glycine max*	XP_003535078.1	0	0.99
GmREV-L-1	*Glycine max*	XP_003538150.1	0	0.99
GmHB14-L-3	*Glycine max*	XP_003532788.1	0	0.99
GmHB14-L-4	*Glycine max*	XP_003524993.1	0	0.99
GsHB8-L-1	*Glycine soja*	KHN10188.1	0	0.98
GsHB8-L-2	*Glycine soja*	KHN18159.1	0	0.98
GsHB8-L-3	*Glycine soja*	RZC15680.1	0	0.98
GsHB15-L-1	*Glycine soja*	RZB97876.1	0	0.99
GsHB15-L-2	*Glycine soja*	RZC00893.1	0	0.99
GsHB15-L-3	*Glycine soja*	KHN39244.1	0	0.99
GsHB15-L-4	*Glycine soja*	KHN39243.1	0	0.99
GsHB15-L-5	*Glycine soja*	XP_028244500.1	0	0.89
GsHB14-L-1	*Glycine soja*	RZB90278.1	0	0.99
GsREV-L-1	*Glycine soja*	XP_028194395.1	0	0.99
GsREV-L-2	*Glycine soja*	KHN38761.1	0	0.99
GsREV-L-3	*Glycine soja*	KHN05682.1	0	0.99
GsHB14-L-2	*Glycine soja*	XP_028232949.1	0	0.99
GsHOX32-L-1	*Glycine soja*	KHN06475.1	0	0.99
GsHOX32-L-2	*Glycine soja*	KHN48072.1	0	0.98
MtrHB8-L-1	*Medicago truncatula*	XP_003603630.2	0	0.98
MtrHB15-L-1	*Medicago truncatula*	XP_003627005.1	0	0.99
MtrHB15-L-2	*Medicago truncatula*	XP_003597690.1	0	0.98
MtrHB14-L-1	*Medicago truncatula*	XP_003594520.2	0	0.98
MtrREV-L-1	*Medicago truncatula*	XP_013456006.1	0	0.99
PvHB8-L-1	*Phaseolus vulgaris*	XP_007137231.1	0	0.98
PvHB15-L-1	*Phaseolus vulgaris*	XP_007150614.1	0	0.98
PvHB15-L-2	*Phaseolus vulgaris*	XP_007135643.1	0	0.99
PvHB14-L-1	*Phaseolus vulgaris*	XP_007147487.1	0	0.99
PvREV-L-1	*Phaseolus vulgaris*	XP_007132262.1	0	0.99
PvHB14-L-2	*Phaseolus vulgaris*	XP_007159548.1	0	0.98

Table S3. Basic physical and chemical information of soybean HD-ZIP III proteins.

Gene ID	Gm HD-ZIP III	Number of amino acids	Theoretical pI	Molecular weight /Da	Instability index	Grand average of hydropathicity	SL
GLYMA_04G085000	GmHB8-L-1	844	6.03	92741.09	47.06	-0.108	nucleus
GLYMA_06G086600	GmHB8-L-2	845	6.03	92923.27	47.63	-0.102	nucleus
GLYMA_08G201900	GmHB15-L-1	838	6.06	92386.59	45.74	-0.141	nucleus
GLYMA_07G016500	GmHB15-L-2	838	6.06	92450.72	45.94	-0.140	nucleus
GLYMA_07G016700	GmHB15-L-3	841	6.12	92947.16	47.68	-0.180	nucleus
GLYMA_08G202000	GmHB15-L-4	843	6.17	93335.68	46.7	-0.180	nucleus
GLYMA_15G129700	GmHB14-L-1	846	5.89	92842.00	51.05	-0.134	nucleus
GLYMA_09G023600	GmHB14-L-2	846	5.89	92779.67	48.46	-0.140	nucleus
GLYMA_08G124400	GmHB14-L-3	849	6.25	93367.67	48.99	-0.115	nucleus
GLYMA_05G166400	GmHB14-L-4	853	6.06	93958.15	50.47	-0.133	nucleus
GLYMA_11G145800	GmREV-L-1	842	5.75	92062.93	47.29	-0.080	nucleus
GLYMA_12G075800	GmREV-L-2	841	5.75	92012.89	47.36	-0.087	nucleus

Table S4. cis-acting elements in the promoter region of GmHD-Zip III family gene.

Gene ID	The bases	Cis acting element
GLYMA_04G085000.1	CTAACGTATT	cis-acting element involved in light responsiveness
GLYMA_04G085000.1	AACGAC	auxin-responsive element
GLYMA_04G085000.1	TGACGTAA	part of an auxin-responsive element
GLYMA_04G085000.1	CACGTG	cis-acting regulatory element involved in light responsiveness
GLYMA_04G085000.1	CACGTT	cis-acting regulatory element involved in light responsiveness
GLYMA_04G085000.1	CACGTG	cis-acting regulatory element involved in light responsiveness
GLYMA_04G085000.1	CACGAC	cis-acting regulatory element involved in light responsiveness
GLYMA_04G085000.1	TACGTG	cis-acting regulatory element involved in light responsiveness
GLYMA_04G085000.1	CGCACGTGTC	cis-acting element involved in the abscisic acid responsiveness
GLYMA_04G085000.1	CACGTG	cis-acting element involved in the abscisic acid responsiveness
GLYMA_04G085000.1	ACGTG	cis-acting element involved in the abscisic acid responsiveness
GLYMA_04G085000.1	ACGTG	cis-acting element involved in the abscisic acid responsiveness
GLYMA_04G085000.1	ACGTG	cis-acting element involved in the abscisic acid responsiveness
GLYMA_04G085000.1	GGTTAA	light responsive element
GLYMA_04G085000.1	CGTCA	cis-acting regulatory element involved in the MeJA-responsiveness
GLYMA_04G085000.1	CGTCA	cis-acting regulatory element involved in the MeJA-responsiveness
GLYMA_04G085000.1	CGTCA	cis-acting regulatory element involved in the MeJA-responsiveness
GLYMA_04G085000.1	CAACTG	MYB binding site involved in drought-inducibility
GLYMA_04G085000.1	CAACTG	MYB binding site involved in drought-inducibility
GLYMA_04G085000.1	CAACTG	MYB binding site involved in drought-inducibility
GLYMA_04G085000.1	ATTCTCTAAC	cis-acting element involved in defense and stress responsiveness
GLYMA_04G085000.1	GTTTTCTTAC	cis-acting element involved in defense and stress responsiveness
GLYMA_04G085000.1	TGACG	cis-acting regulatory element involved in the MeJA-responsiveness
GLYMA_04G085000.1	TGACG	cis-acting regulatory element involved in the MeJA-responsiveness
GLYMA_04G085000.1	TGACG	cis-acting regulatory element involved in the MeJA-responsiveness
GLYMA_04G085000.1	TCTGTTG	gibberellin-responsive element
GLYMA_04G085000.1	CCGAAA	cis-acting element involved in low-temperature responsiveness
GLYMA_04G085000.1	GGTCCAT	cis-acting regulatory element involved in auxin responsiveness
GLYMA_05G166400.1	CAACTG	MYB binding site involved in drought-inducibility
GLYMA_05G166400.1	CAACTG	MYB binding site involved in drought-inducibility
GLYMA_05G166400.1	CAACTG	MYB binding site involved in drought-inducibility
GLYMA_05G166400.1	CAACTG	MYB binding site involved in drought-inducibility
GLYMA_05G166400.1	CAACTG	MYB binding site involved in drought-inducibility
GLYMA_05G166400.1	CGTCA	cis-acting regulatory element involved in the MeJA-responsiveness
GLYMA_05G166400.1	CCGAAA	cis-acting element involved in low-temperature responsiveness
GLYMA_05G166400.1	TGACG	cis-acting regulatory element involved in the MeJA-responsiveness
GLYMA_05G166400.1	AACGAC	auxin-responsive element
GLYMA_05G166400.1	TCTTAC	part of a light responsive element
GLYMA_05G166400.1	TCTTAC	part of a light responsive element
GLYMA_05G166400.1	CACGAC	cis-acting regulatory element involved in light responsiveness
GLYMA_06G086600.1	CACGTG	cis-acting element involved in the abscisic acid responsiveness
GLYMA_06G086600.1	ACGTG	cis-acting element involved in the abscisic acid responsiveness
GLYMA_06G086600.1	ACGTG	cis-acting element involved in the abscisic acid responsiveness
GLYMA_06G086600.1	CACGTG	cis-acting regulatory element involved in light responsiveness
GLYMA_06G086600.1	CACGAC	cis-acting regulatory element involved in light responsiveness
GLYMA_06G086600.1	CACGTG	cis-acting regulatory element involved in light responsiveness
GLYMA_06G086600.1	CACGTT	cis-acting regulatory element involved in light responsiveness
GLYMA_06G086600.1	AACGAC	auxin-responsive element
GLYMA_06G086600.1	TGACGTAA	part of an auxin-responsive element

GLYMA_06G086600.1	CTAACGTATT	cis-acting element involved in light responsiveness
GLYMA_06G086600.1	CCGAAA	cis-acting element involved in low-temperature responsiveness
GLYMA_06G086600.1	GGTCCAT	cis-acting regulatory element involved in auxin responsiveness
GLYMA_06G086600.1	GGGCGG	light responsive element
GLYMA_06G086600.1	TGACG	cis-acting regulatory element involved in the MeJA-responsiveness
GLYMA_06G086600.1	TGACG	cis-acting regulatory element involved in the MeJA-responsiveness
GLYMA_06G086600.1	TGACG	cis-acting regulatory element involved in the MeJA-responsiveness
GLYMA_06G086600.1	TGACG	cis-acting regulatory element involved in the MeJA-responsiveness
GLYMA_06G086600.1	TGACG	cis-acting regulatory element involved in the MeJA-responsiveness
GLYMA_06G086600.1	TCTGTTG	gibberellin-responsive element
GLYMA_06G086600.1	TCTGTTG	gibberellin-responsive element
GLYMA_06G086600.1	CAACTG	MYB binding site involved in drought-inducibility
GLYMA_06G086600.1	CAACTG	MYB binding site involved in drought-inducibility
GLYMA_06G086600.1	ATTCTCTAAC	cis-acting element involved in defense and stress responsiveness
GLYMA_06G086600.1	GTTTTCTTAC	cis-acting element involved in defense and stress responsiveness
GLYMA_06G086600.1	CGTCA	cis-acting regulatory element involved in the MeJA-responsiveness
GLYMA_06G086600.1	CGTCA	cis-acting regulatory element involved in the MeJA-responsiveness
GLYMA_06G086600.1	CGTCA	cis-acting regulatory element involved in the MeJA-responsiveness
GLYMA_06G086600.1	CGTCA	cis-acting regulatory element involved in the MeJA-responsiveness
GLYMA_06G086600.1	CGTCA	cis-acting regulatory element involved in the MeJA-responsiveness
GLYMA_07G016500.1	CGTCA	cis-acting regulatory element involved in the MeJA-responsiveness
GLYMA_07G016500.1	CGTCA	cis-acting regulatory element involved in the MeJA-responsiveness
GLYMA_07G016500.1	CGTCA	cis-acting regulatory element involved in the MeJA-responsiveness
GLYMA_07G016500.1	CGTCA	cis-acting regulatory element involved in the MeJA-responsiveness
GLYMA_07G016500.1	CGTCA	cis-acting regulatory element involved in the MeJA-responsiveness
GLYMA_07G016500.1	CAACTG	MYB binding site involved in drought-inducibility
GLYMA_07G016500.1	TGACG	cis-acting regulatory element involved in the MeJA-responsiveness
GLYMA_07G016500.1	TGACG	cis-acting regulatory element involved in the MeJA-responsiveness
GLYMA_07G016500.1	TGACG	cis-acting regulatory element involved in the MeJA-responsiveness
GLYMA_07G016500.1	TGACG	cis-acting regulatory element involved in the MeJA-responsiveness
GLYMA_07G016500.1	TGACG	cis-acting regulatory element involved in the MeJA-responsiveness
GLYMA_07G016500.1	AGAAACTT	part of a module for light response
GLYMA_07G016500.1	AGTAATCT	part of a conserved DNA module involved in light responsiveness
GLYMA_07G016700.1	CACGTC	cis-acting regulatory element involved in light responsiveness
GLYMA_07G016700.1	ACGTG	cis-acting element involved in the abscisic acid responsiveness
GLYMA_07G016700.1	TGACG	cis-acting regulatory element involved in the MeJA-responsiveness
GLYMA_07G016700.1	TGACG	cis-acting regulatory element involved in the MeJA-responsiveness
GLYMA_07G016700.1	TGACG	cis-acting regulatory element involved in the MeJA-responsiveness
GLYMA_07G016700.1	TGACG	cis-acting regulatory element involved in the MeJA-responsiveness
GLYMA_07G016700.1	AGAAACTT	part of a module for light response
GLYMA_07G016700.1	AACCTAA	MYB binding site involved in light responsiveness
GLYMA_07G016700.1	CGTCA	cis-acting regulatory element involved in the MeJA-responsiveness
GLYMA_07G016700.1	CGTCA	cis-acting regulatory element involved in the MeJA-responsiveness
GLYMA_07G016700.1	CGTCA	cis-acting regulatory element involved in the MeJA-responsiveness
GLYMA_07G016700.1	CGTCA	cis-acting regulatory element involved in the MeJA-responsiveness
GLYMA_07G016700.1	CAACTG	MYB binding site involved in drought-inducibility
GLYMA_08G124400.1	AGAAACTT	part of a module for light response
GLYMA_08G124400.1	TGACG	cis-acting regulatory element involved in the MeJA-responsiveness
GLYMA_08G124400.1	TGACG	cis-acting regulatory element involved in the MeJA-responsiveness
GLYMA_08G124400.1	CAACTG	MYB binding site involved in drought-inducibility
GLYMA_08G124400.1	CAACTG	MYB binding site involved in drought-inducibility

GLYMA_08G124400.1	CAACTG	MYB binding site involved in drought-inducibility
GLYMA_08G124400.1	CAACTG	MYB binding site involved in drought-inducibility
GLYMA_08G124400.1	CAACTG	MYB binding site involved in drought-inducibility
GLYMA_08G124400.1	CGTCA	cis-acting regulatory element involved in the MeJA-responsiveness
GLYMA_08G124400.1	CGTCA	cis-acting regulatory element involved in the MeJA-responsiveness
GLYMA_08G124400.1	TCTTAC	part of a light responsive element
GLYMA_08G124400.1	TCTTAC	part of a light responsive element
GLYMA_08G124400.1	CACGAC	cis-acting regulatory element involved in light responsiveness
GLYMA_08G124400.1	AACGAC	auxin-responsive element
GLYMA_08G201900.1	AGAAACTT	part of a module for light response
GLYMA_08G201900.1	TGACG	cis-acting regulatory element involved in the MeJA-responsiveness
GLYMA_08G201900.1	TGACG	cis-acting regulatory element involved in the MeJA-responsiveness
GLYMA_08G201900.1	TGACG	cis-acting regulatory element involved in the MeJA-responsiveness
GLYMA_08G201900.1	TGACG	cis-acting regulatory element involved in the MeJA-responsiveness
GLYMA_08G201900.1	CAACTG	MYB binding site involved in drought-inducibility
GLYMA_08G201900.1	CGTCA	cis-acting regulatory element involved in the MeJA-responsiveness
GLYMA_08G201900.1	CGTCA	cis-acting regulatory element involved in the MeJA-responsiveness
GLYMA_08G201900.1	CGTCA	cis-acting regulatory element involved in the MeJA-responsiveness
GLYMA_08G201900.1	CGTCA	cis-acting regulatory element involved in the MeJA-responsiveness
GLYMA_08G202000.1	AGAAACTT	part of a module for light response
GLYMA_08G202000.1	TGACG	cis-acting regulatory element involved in the MeJA-responsiveness
GLYMA_08G202000.1	TGACG	cis-acting regulatory element involved in the MeJA-responsiveness
GLYMA_08G202000.1	TGACG	cis-acting regulatory element involved in the MeJA-responsiveness
GLYMA_08G202000.1	TGACG	cis-acting regulatory element involved in the MeJA-responsiveness
GLYMA_08G202000.1	TGACG	cis-acting regulatory element involved in the MeJA-responsiveness
GLYMA_08G202000.1	GGGCGG	light responsive element
GLYMA_08G202000.1	AACCTAA	MYB binding site involved in light responsiveness
GLYMA_08G202000.1	CGTCA	cis-acting regulatory element involved in the MeJA-responsiveness
GLYMA_08G202000.1	CGTCA	cis-acting regulatory element involved in the MeJA-responsiveness
GLYMA_08G202000.1	CGTCA	cis-acting regulatory element involved in the MeJA-responsiveness
GLYMA_08G202000.1	CGTCA	cis-acting regulatory element involved in the MeJA-responsiveness
GLYMA_08G202000.1	CGTCA	cis-acting regulatory element involved in the MeJA-responsiveness
GLYMA_08G202000.1	GGTTAAT	light responsive element
GLYMA_08G202000.1	ACGTG	cis-acting element involved in the abscisic acid responsiveness
GLYMA_08G202000.1	ACGTG	cis-acting element involved in the abscisic acid responsiveness
GLYMA_08G202000.1	TACGTG	cis-acting regulatory element involved in light responsiveness
GLYMA_08G202000.1	CACGTC	cis-acting regulatory element involved in light responsiveness
GLYMA_08G202000.1	AACGAC	auxin-responsive element
GLYMA_09G023600.1	CACGTG	cis-acting element involved in the abscisic acid responsiveness
GLYMA_09G023600.1	ACGTG	cis-acting element involved in the abscisic acid responsiveness
GLYMA_09G023600.1	TCTCCCT	part of a light responsive element
GLYMA_09G023600.1	CACGAC	cis-acting regulatory element involved in light responsiveness
GLYMA_09G023600.1	CACGAC	cis-acting regulatory element involved in light responsiveness
GLYMA_09G023600.1	CACGAC	cis-acting regulatory element involved in light responsiveness
GLYMA_09G023600.1	CACGTG	cis-acting regulatory element involved in light responsiveness
GLYMA_09G023600.1	CACGTG	cis-acting regulatory element involved in light responsiveness
GLYMA_09G023600.1	CTTTATCA	part of a light responsive element
GLYMA_09G023600.1	TCTGTTG	gibberellin-responsive element
GLYMA_09G023600.1	TCTGTTG	gibberellin-responsive element
GLYMA_09G023600.1	CCGAAA	cis-acting element involved in low-temperature responsiveness
GLYMA_09G023600.1	CAACTG	MYB binding site involved in drought-inducibility

GLYMA_09G023600.1	CAACTG	MYB binding site involved in drought-inducibility
GLYMA_11G145800.1	CAACTG	MYB binding site involved in drought-inducibility
GLYMA_11G145800.1	CAACTG	MYB binding site involved in drought-inducibility
GLYMA_11G145800.1	TCTCACCAACCACA	light responsive element
GLYMA_11G145800.1	CGTCA	cis-acting regulatory element involved in the MeJA-responsiveness
GLYMA_11G145800.1	CCTTTTG	gibberellin-responsive element
GLYMA_11G145800.1	AACCTAA	MYB binding site involved in light responsiveness
GLYMA_11G145800.1	TAAGAGAGGAA	light responsive element
GLYMA_11G145800.1	AGAAACAA	part of a module for light response
GLYMA_11G145800.1	GATAGGA	part of a light responsive element
GLYMA_11G145800.1	TGACG	cis-acting regulatory element involved in the MeJA-responsiveness
GLYMA_11G145800.1	TATCCCA	cis-acting element involved in gibberellin-responsiveness
GLYMA_11G145800.1	GACACGTGGC	cis-acting element involved in the abscisic acid responsiveness
GLYMA_11G145800.1	TCTCCCT	part of a light responsive element
GLYMA_11G145800.1	TCTTAC	part of a light responsive element
GLYMA_11G145800.1	TCTTAC	part of a light responsive element
GLYMA_15G129700.1	CAACAAACCCCTT	gibberellin-responsive element and part of a light responsive element
GLYMA_15G129700.1	CAACTG	MYB binding site involved in drought-inducibility
GLYMA_15G129700.1	CAACTG	MYB binding site involved in drought-inducibility
GLYMA_15G129700.1	CAACTG	MYB binding site involved in drought-inducibility
GLYMA_15G129700.1	CAACTG	MYB binding site involved in drought-inducibility
GLYMA_15G129700.1	TCTGTTG	gibberellin-responsive element
GLYMA_15G129700.1	CTTTATCA	part of a light responsive element
GLYMA_15G129700.1	TCTTAC	part of a light responsive element
GLYMA_15G129700.1	CACGAC	cis-acting regulatory element involved in light responsiveness
GLYMA_15G129700.1	CACGAC	cis-acting regulatory element involved in light responsiveness
GLYMA_15G129700.1	CACGAC	cis-acting regulatory element involved in light responsiveness
